# Perceived Anger in Clear and Conversational Speech: Contributions of Age and Hearing Loss

**DOI:** 10.3390/brainsci12020210

**Published:** 2022-02-02

**Authors:** Shae D. Morgan, Sarah Hargus Ferguson, Ashton D. Crain, Skyler G. Jennings

**Affiliations:** 1 Department of Otolaryngology—Head and Neck Surgery and Communicative Disorders, University of Louisville, Louisville, KY 40241, USA; 2 Department of Communication Sciences and Disorders, University of Utah, Salt Lake City, UT 84111, USA; sarah.ferguson@hsc.utah.edu (S.H.F.); a.crain1991@gmail.com (A.D.C.); skyler.jennings@hsc.utah.edu (S.G.J.)

**Keywords:** clear speech, conversational speech, perceived emotion, aging, hearing loss

## Abstract

A previous investigation demonstrated differences between younger adult normal-hearing listeners and older adult hearing-impaired listeners in the perceived emotion of clear and conversational speech. Specifically, clear speech sounded angry more often than conversational speech for both groups, but the effect was smaller for the older listeners. These listener groups differed by two confounding factors, age (younger vs. older adults) and hearing status (normal vs. impaired). The objective of the present study was to evaluate the contributions of aging and hearing loss to the reduced perception of anger in older adults with hearing loss. We investigated perceived anger in clear and conversational speech in younger adults with and without a simulated age-related hearing loss, and in older adults with normal hearing. Younger adults with simulated hearing loss performed similarly to normal-hearing peers, while normal-hearing older adults performed similarly to hearing-impaired peers, suggesting that aging was the primary contributor to the decreased anger perception seen in previous work. These findings confirm reduced anger perception for older adults compared to younger adults, though the significant speaking style effect—regardless of age and hearing status—highlights the need to identify methods of producing clear speech that is emotionally neutral or positive.

## 1. Introduction

Talkers may adopt a more clear speaking style to overcome perceived barriers to communication (e.g., when the listener has a hearing loss or is a non-native speaker of the talker’s language). This clear speaking style serves to improve the communicative experience by affording the listener a speech-understanding benefit compared to when a more conversational style is used (e.g., [1,2]). Clear speech has been found to be associated with numerous acoustic changes from habitual/conversational speech, which are thought to result in this clear speech benefit [3]. At the suprasegmental level, these changes include raised and more variable voice fundamental frequency, decreased speaking rate, increased energy in the 1000–3000 Hz range of the long-term speech spectrum, and larger fluctuations in the temporal envelope [4,5]. At the segmental level, stop consonants are released more often [5,6], voice onset time of word-initial voiceless stop consonants increases [5], and short-term vowel spectra change in a manner that expands the acoustic vowel space [7,8].

While adopting these segmental and suprasegmental modifications makes speech easier to understand for many different listener groups, there has been some speculation recently about other, unintended consequences of clear speech, such as the increased perception of anger [9]. Indeed, many of the suprasegmental acoustic features found in clear speech, including increased high-frequency energy and greater pitch variability, have also been reported in angry speech [10,11]. Thus, the pattern of acoustic modifications that talkers use to make themselves easier to understand may also promote the perception of anger and other negative emotions.

Directly investigating perceived anger in clear and conversational speech, Morgan and Ferguson [9] found that both younger adults with normal hearing and older adults with hearing loss rated clear speech as sounding angry more often than conversational speech. The older adult group with hearing loss, however, showed generally reduced emotion perception, opting to choose “neutral” more often than younger adults with normal hearing. As these listener groups differed primarily on two variables, age and hearing status, it was impossible for the authors to infer the source of this group difference from their data. The following sections will review auditory emotion recognition, and propose how aging and hearing loss may result in the reduced perception of anger observed in clear and conversational speech.

### 1.1. Emotion Recognition

In psychological sciences, emotion is generally described using one of three models: discrete [12], dimensional [13], or a combination of the two. For the purposes of this research, we will focus on a discrete model, where emotions are thought to have distinct categories [12,14]. Discrete models are considered the simplest for describing emotions, as they use basic labels to categorize emotions, such as happiness, sadness, fear, anger, disgust, contempt, and surprise. According to Ekman and Cordaro [14], these seven emotions are general enough to be cross-cultural, and therefore are appropriate for use across generations.

### 1.2. Effect of Age on Emotion Recognition

Aging differences have been observed in the emotional perception of both visual and acoustic stimuli [15,16], suggesting that reduced perception of negative emotions is associated with aging. Some groups have even named this observation the “positivity effect”, though the robustness and universality of that effect is in question [17]. These differences are thought to be caused by sociocognitive and/or neuropsychological factors. Sociocognitive explanations suggest that aging is accompanied by an increased ability to understand and regulate emotions. This is attributed to extensive life experience in analyzing emotional cues [18]. Neuropsychological explanations, in contrast, posit that the aging process contributes to decreased activity in the brain regions responsible for emotion processing and regulation. Compared to visual perception, these neuropsychological accounts are less well-documented in auditory perception of emotion (e.g., [19]).

A recent study on auditory emotion recognition across the lifespan suggests that emotion recognition performance improves and approaches adult-like levels in early adolescence [20]. Early adulthood marks a stable period of auditory emotion recognition ability, followed by a gradual decline into later adulthood. Older adults (>60 years old) showed a sharp reduction in the accuracy of emotional identification. This may be explained by socioemotional selectivity theory, which suggests that older adults tend to neutralize (or even positively valence) their perceptions of the emotions of others. Another explanation is that older adults may require more dramatic examples of emotions to warrant classification as such compared to younger adults, who are more attentive to the subtle emotionality needed to advance through adulthood (e.g., to attract a partner, secure advancement at work, etc.). It is also possible, however, that declines in hearing contribute to emotion perception in older adults. These declines include reduced access to and processing of high-frequency acoustic cues [21], diminished temporal processing [22], and age-related loss of auditory nerve fibers [23].

### 1.3. Effect of Hearing Loss on Emotion Recognition

The effect of hearing loss on auditory emotion recognition is an expanding area of research interest. Recent studies have shown conflicting reports, with some suggesting no relationship between hearing loss and emotion recognition ability (e.g., [24]), while others show clear associations [25,26,27,28,29]. Investigations involving participants with mild-to-moderate hearing loss usually find minimal or no reduction of emotion recognition when compared with typically hearing peers. In contrast, studies centering on individuals with more severe hearing loss, and especially individuals with cochlear implants, show clear deficits. There seems to be a critical threshold of hearing loss required to significantly impact the ability to access emotional content from auditory stimuli. Older adults naturally tend to have more significant and severe hearing losses than younger adults, and it may be that this hearing loss is significant enough to reduce older adults’ access to the acoustic cues associated with different emotion categories. For example, one acoustic feature of angry speech is increased high-frequency energy compared to emotionally neutral speech (e.g., [10]). An inability to discern this increased energy (due to hearing loss at high frequencies) may result in reduced anger perception for individuals with hearing loss compared to peers with normal hearing.

### 1.4. Previous Research

In this manuscript we present two experiments that, when compared with previously published data from our research group [9], provide additional insight into the independent contributions of aging and hearing status for the perception of anger in clear and conversational speech. These experiments include (1) assessing the effects of a simulated hearing loss on emotion perception in younger adults with normal hearing, and (2) comparing emotion perception among older adults with normal hearing and hearing loss. We hypothesized that both aging (through sociocognitive and neuropsychological changes) and hearing loss (through reduced sensitivity to acoustic cues that typify emotional productions) would contribute to the overall reduction in the perception of anger for older adults with hearing loss when listening to clear and conversational speech. Understanding which of these contributions is dominant will help determine when using clear speech carries the greatest risk of invoking a negative sentiment. If aging better explains reduced anger perception than hearing loss, then hearing health professionals should take extra care when counseling younger patients with hearing loss (a primary population to whom clear speech is directed) to be wary of unintended negativity when communication partners use this speaking style. On a more foundational level, the present study also provides critical insight regarding the primary mechanisms surrounding emotional perception (i.e., cognitive mechanisms versus peripheral sensitivity) and their independent or combined contributions.

## 2. Materials and Methods

### 2.1. Experiment I: Young Adults with a Simulated Hearing Loss

The objective of this experiment was to determine the extent to which the reduced audibility of high frequencies, independent of age, affects the perceived emotion of clear and conversational speech by simulating hearing loss in young adults with normal hearing.

#### 2.1.1. Stimuli

Speech stimuli used in this experiment came from the Ferguson Clear Speech Database (described in [30]). The database consists of 41 talkers, from whom the same list of 188 sentences were recorded in clear and conversational speaking styles. To reduce semantic priming of specific emotions, 14 emotionally neutral sentences were chosen for the current study (e.g., “Use the word bead in a sentence”). In total, 8 talkers producing these 14 sentences in each speaking style were selected from the database for the current study (2 speaking styles × 8 talkers × 14 sentences = 224 total stimuli). The talkers were combined into two groups based on their perceived clarity (see [9] for additional details): Group A and Group B. Talkers in Group A were rated to have the largest clear speech effect and were considered “good” clear speakers, while the talkers in Group B had the smallest clear speech effect and were considered “poor” clear speakers.

**Figure 1:**
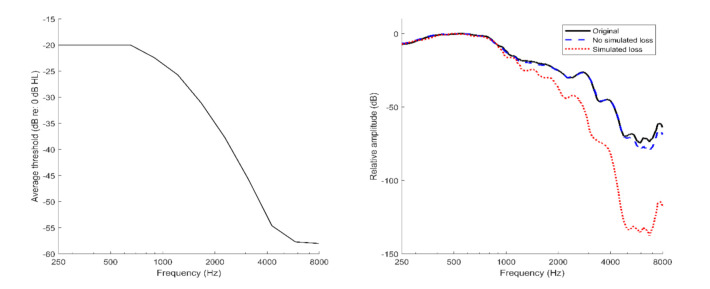
Average simulated hearing loss based on the older adult data from Morgan and Ferguson [9] (left) and the long-term average speech spectrum of 30 s samples of concatenated stimuli from the original unprocessed stimuli, the stimuli processed with no simulated loss, and the stimuli processed with the simulated hearing loss.

We processed the stimuli used in Morgan and Ferguson [9] to simulate aspects of age-related hearing loss following methods and procedures described by Moore and Glasberg [31]. Their approach accounts for the effects of reduced sensitivity and abnormal loudness growth across a bank of simulated auditory filters. Specifically, the following processing steps were completed: (1) split the stimulus into frequency bands; (2) isolate the temporal envelope and fine structure within each band; (3) apply an exponent to the envelope to simulate envelope expansion and loudness recruitment; (4) smooth the expanded envelope of each band; (5) calculate and apply the attenuation level for each band; and (6) sum across bands after recombining and temporally aligning the processed envelope and fine structure. Figure 1 shows the attenuation applied to each frequency band (based on the average audiogram of the older adults with hearing loss from Morgan and Ferguson [9]), as well as the realization of that attenuation (following procedures by Moore and Glasberg [31]) in the long-term average spectra for the original and unprocessed stimuli (created using a 30 s sample of concatenated stimuli from each condition and calculating the fast-Fourier transform via the *fft* function in MATLAB). Similarly, according to Moore and Glasberg, the degree of envelope expansion was directly determined by the attenuation level of each band. The simulation of envelope expansion accounts for loss of cochlear compression; however, such expansion may also occur within the central auditory system due to increased central gain [32]. Our simulations are limited to accounting for aspects of age-related hearing loss that are attributed to changes in the auditory periphery (i.e., reduced audibility and loss of cochlear compression). Thus, any effects of hearing loss attributed to central auditory function (e.g., [33]) are not accounted for by the model. Despite this, our simulations address our hypothesis that age-related changes in emotion perception may be due to the reduced audibility of the acoustic cues that mark a talker’s emotion.

In addition to processing the stimuli to simulate hearing loss, we included a control set of stimuli, which underwent the same processing (i.e., band-pass filtering, envelope expansion, etc.) as the experimental stimuli, except no attenuation was applied to the frequency bands (effectively simulating hearing thresholds at 0 dB HL). The stimuli were scaled to the same average root-mean-square amplitude in Cool Edit 2000 to limit the use of amplitude differences as an emotional cue between the speaking styles; this procedure is common in clear speech perception research [34].

#### 2.1.2. Listeners

A total of 44 younger adults (18 to 32 years old; M = 21, SD = 3.2) with normal hearing were recruited for this experiment. Normal hearing was established via a pure tone screening at the time of testing, with all participants demonstrating thresholds better than or equal to 20 dB HL at octave intervals from 250 to 8000 Hz. These participants were divided into two groups (22 listeners per group) that were similar in their average age and hearing thresholds. One group rated the processed control stimuli (i.e., without high-frequency attenuation) and the other group rated the processed experimental stimuli (i.e., with high-frequency attenuation; YSIM). All listeners in this experiment passed the Montreal Cognitive Assessment [35]. All listeners denied any history of speech or language disorders or therapy and were native speakers of American English.

#### 2.1.3. Procedures

All subject recruitment and other procedures were in accordance with a protocol approved by the University of Utah Institutional Review Board. Participants sat in a sound-treated room in front of a computer monitor, mouse, and keyboard. Test methods were similar to those used in Morgan and Ferguson [9]. A custom MATLAB graphical user interface (GUI) presented the instructions and guided the listeners through the experiment. Instructions were provided verbally and on screen. Participants were told, “Judge the emotion you think you hear when listening to each sentence.” The emotional category options were “anger,” “fear,” “disgust,” “sadness,” “happiness,” and “neutral,” presented in a six-alternative, forced-choice task paradigm. Participants were instructed to choose “neutral” if they heard no specific emotion in the speech. Ekman and Cordaro [14] recommend these emotions as “basic” emotion categories. Contempt and surprise are also considered “basic” emotions, but they were excluded from this study. Contempt was excluded because it is often difficult to conceptualize due to its lack of common use [36], and surprise was excluded because it is a brief emotion, quickly shifting to another emotion immediately after its expression [14].

Stimuli were presented through a Tucker-Davis Technologies (TDT) RP2.1 real-time processor and then attenuated by a TDT programmable attenuator (PA-5) to a comfortable listening level (70 dB SPL using a 1 kHz calibration tone). The stimuli were then routed via a headphone buffer (TDT HB7) to Sennheiser HD 515 headphones for monaural presentation to the participant’s test ear, which was selected by the participant as their perceived better ear. Monaural presentation was chosen to mimic procedures used for the older adult group with hearing loss (OHL) in Morgan and Ferguson [9]. Task familiarization consisted of 20 items that were presented in a practice block prior to testing. All participants confirmed that they felt confident with the task after completing the practice block. For the experiment, listeners heard 224 sentences in a random order without replacement. Participants responded by clicking on the emotion category they felt best corresponded to the sentence and then pressed “Enter” on the keyboard to advance to the next presentation. Listeners were given the opportunity to listen to each stimulus one additional time by clicking a button in the GUI labeled “Listen again”. Listeners were offered breaks at regular intervals during each test block.

#### 2.1.4. Statistical Analysis

The responses were combined across the listeners in each group to create a dependent variable of the percentage of listeners who judged a given stimulus to be angry. These data were then analyzed using a linear mixed-effects models via the *lme4* [37] and *lmerTest* [38] packages in *R* (version 3.5.1; [39]). In each model, the fixed effects of speaking style (clear and conversational), listener group (YNH, OHL, and YSIM), and talker group (A and B) were analyzed, along with all two- and three-way interactions among them, and talker was included as a random factor. Main effects were assessed using the *anova* function in *lmerTest*, which implements the Satterthwaite approximation for degrees of freedom to calculate *p*-values [40]. In contrast to using likelihood ratio tests (another popular method for assessing significance of variables in linear mixed-effects models), this approach has been shown to yield more conservative estimates that are less prone to Type I errors [41].

A data integrity check comparing data obtained from YNH listeners in Morgan and Ferguson [9] and the processed control data (with no applied attenuation) from this study showed no significant fixed or interaction effects involving listener group (all *p* > 0.26). This confirmed that any effects related to the simulated hearing loss are a result of the attenuation and recruitment simulation and not the other aspects of the processing.

#### 2.1.5. Results

**Figure 2:**
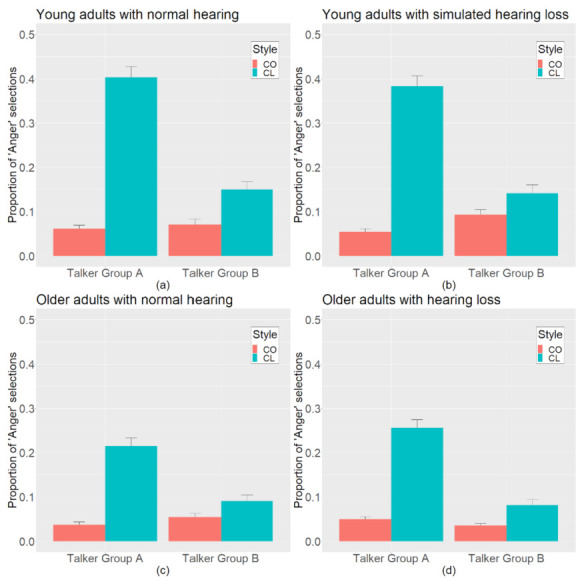
Proportion of “Anger” selections for clear (CL) and conversational (CO) speech by (**a**) young adults with normal hearing; YNH, (**b**) young adults with a simulated hearing loss; YSIM, (**c**) older adults with normal hearing; ONH, and (**d**) older adults with hearing loss; OHL. Error bars represent one standard error.

Figure 2 shows the perceived anger in clear and conversational speech for the two talker groups and the three listener groups (panels a, b, and d). Statistical analyses showed a significant main effect of talker (*F*_(1, 658)_ = 17.4, *p* < 0.001), a significant interaction between talker and speaking style (*F*_(1, 658)_ = 129.5, *p* < 0.001), and a significant three-way interaction between speaking style, talker group, and listener group (*F*_(1, 658)_ = 6.9, *p* < 0.001). In general, clear speech was rated as sounding angry more often than conversational speech, and that effect was more pronounced for “good” clear speakers than for the “poor” clear speakers in this study. The nature of the three-way interaction can be seen in Figure 2, which shows a greater interaction between speaking style and talker group for the YNH and YSIM groups (which appear to be more similar) than the ONH group (which has a smaller interaction relative to the other two groups).

### 2.2. Experiment II: Older Adults with Essentially Normal Hearing

Experiment I showed no significant effect of simulated hearing loss on the perception of anger in clear and conversational speech in a group of young adult listeners. This finding suggests that the reduced perception of anger in clear and conversational speech in older adults with hearing loss is driven by the effects of age, rather than the effects of hearing sensitivity. To confirm this, in Experiment II we repeated the study/task of auditory emotion perception for unprocessed clear and conversational speech materials with a sample of older adults with normal or borderline normal hearing (ONH; described below).

#### 2.2.1. Stimuli

Stimuli for Experiment II were the same as Experiment I, except the stimuli were not processed to simulate hearing loss or produce control stimuli. These unprocessed stimuli were the same as those used in Morgan and Ferguson [9].

#### 2.2.2. Listeners

A total of 17 older adult listeners (66 to 83 years old; M = 72.77, SD 4.47) were recruited from the Utah Senior Ears database. This database recruits community members who are interested in participating in hearing-related research in exchange for periodic hearing tests at no charge. The listeners in this study had pure tone thresholds less than or equal to 25 dB HL for 250–3000 Hz and no greater than 40 dB HL for 4000 Hz.

In addition to these hearing status criteria, all listeners in this experiment were administered the Montreal Cognitive Assessment [35]. Two participants in this study did not pass the cognitive screening due to deficits in delayed recall (*n* = 1) or visuospatial processing (*n* = 1). However, data from these participants were included in the data analysis, as there was no significant difference found when their data were excluded. All listeners denied any history of speech or language disorders or therapy and were native speakers of American English. Participants were either compensated for their time (*n* = 6) or opted to participate as volunteers (*n* = 11).

#### 2.2.3. Procedures

The experimental methods and procedures for Experiment II were the same as those used for Experiment I, except the stimuli used in this experiment were not processed to simulate hearing loss.

#### 2.2.4. Statistical Analysis

In total, 17 listeners judged 224 sentences, resulting in 3808 data points for ONH listeners. A total of 121 missing data points occurred due to an experiment software error encountered by some participants which resulted in the premature termination of the experiment (*n* = 6). The data were analyzed in the same manner detailed for Experiment I, but comparing YNH and OHL data with ONH participants rather than YSIM participants from Experiment I.

#### 2.2.5. Results

In addition to the three listener groups from Experiment I, Figure 2 also shows the perceived anger in clear and conversational speech for the two talker groups by ONH listeners (panel c) Statistical analyses revealed a significant main effect of talker (*F*_(1, 658)_ = 17.4, *p* < 0.001), a significant interaction between talker and speaking style (*F*_(1, 658)_ = 98.1, *p* < 0.001), and a significant three-way interaction between speaking style, talker group, and listener group (*F*_(1, 658)_ = 10.4, *p* < 0.01). The nature of this three-way interaction can be seen in Figure 2. The interaction between speaking style and talker group was greater for the YNH group than for the ONH and OHL groups, which appear to be more similar, but with reduced overall perception of anger compared to the YNH group.

Finally, a confirmatory analysis showed a significant listener group effect between YSIM participants in Experiment I and ONH participants in Experiment II (*F*_(1, 434)_ = 4.2, *p* < 0.05), where YSIM participants (Figure 2b) reported hearing anger more often than the ONH group (Figure 2c). In combination with the significant three-way interactions involving the listener group, this analysis confirms that any reduced perception of anger between participant groups is primarily driven by age and not by the hearing status of the participants. Thus, older adults, regardless of hearing status, reported speech as “sounding angry” less often than younger adults, even when younger adults listened to stimuli filtered to simulate the high-frequency hearing loss. The results of this study support the conclusions of Experiment I, which suggested that the differences between the YNH and OHL listeners in Morgan and Ferguson (2017) were due to age effects and not to differences in signal audibility.

## 3. General Discussion

The compiled results from the two experiments presented here and the two experiments presented in Morgan and Ferguson [9] reveal that younger adults, regardless of hearing status (YNH and YSIM listeners) were similar in how often they judged clear speech to sound angry, but that older adults (ONH and OHL listeners), whose ratings were similar regardless of hearing status, rated clear speech as sounding angry less often than either of the younger adult groups. Thus, it appears that the presence or simulation of mild to moderate hearing loss did not significantly affect listeners’ perceived anger in clear and conversational speech, confirming that hearing loss is not the primary contributing factor underlying age-related changes to the perception of anger in clear and conversational speech. Similarities and differences among the younger and older listener groups were found to be larger for clear speech, but these patterns were also observed in ratings of conversational speech, suggesting that even habitual speech is differentially perceived across a person’s lifespan.

Ruffman et al. [15] found that older adults had poorer performance on emotion recognition tasks than younger adults. Older adults in this study perceived anger in vocal expressions less often when compared to younger adults—that is, older adults perceived stimuli to be neutral more often than younger adults. Anatomically, there has been evidence that these findings may be at least partially explained by a consistent age-related decline of the orbitofrontal cortex (one of the crucial areas of the brain related to recognition of anger, sadness, and fearful expressions; [19]). This research provides some general support for the idea that some of the neurological regions involved in emotion recognition are also involved in recognizing facial, auditory, and bodily expressions, and that these regions experience an age-related decline [42].

The discussion thus far has characterized the group differences in terms of age-related decline in emotion recognition. However, sociocognitive theories propose that with aging, one’s ability to understand and regulate emotions actually improves [43]. This suggests that the decrease in perceived anger observed in the current experiments may actually be a result of more accurate perception of the stimuli, which were originally recorded as neutral speech with no emotional intention. Older adults have had a lifetime of analyzing emotional cues in interpersonal communication, and it has been assumed that this skill might be preserved or even improve with aging [44].

### Clinical Implications

The results from this study, as well as supporting evidence from previous studies, confirm that listeners generally judge clear speech as angry more often than conversational speech, regardless of age and hearing status. Clinical audiologists and other health professionals often counsel communication partners of their hearing-impaired patients to adopt a clear speaking style to improve their partner’s speech understanding. The findings of these studies indicate that talkers using this style of speaking may give the impression that the talker is upset, resulting in negative emotions accompanying the improved clarity of speech. This negative perception of clear speech may cause excess stress on relationships for individuals with hearing loss and their communication partners, relationships which are already negatively affected by hearing loss (e.g., [45]). This added stress may have negative social effects on both the hearing-impaired individual and their communication partners. Thus, communication professionals should make these recommendations with caution, particularly for younger adult patients who may be more likely to rate clear speech negatively. While clear speech will likely improve understanding, it may come with unwanted emotional undertones and perceived anger.

## 4. Conclusions

The two experiments detailed in this manuscript confirm that the differences in perceived anger in clear and conversational speech between YNH and OHL listeners are primarily an effect of aging rather than decreased peripheral sensitivity to emotional acoustic cues. Listeners continued to perceive clear speech as angry more often than conversational speech. This finding is consistent with previous research in the emotional perception of clear speech, as well as clinical patient reports. In addition, anger was perceived more often in the clear speaking style with talkers with good clear speech, compared to talkers who had poor clear speech. YNH and YSIM listeners had a larger perception of anger in clear speech when compared to OHL and ONH listeners. These findings support aging as the primary mechanism in reduced perception of anger in clear speech.

Future research could target other listener populations, such as adolescents and middle-aged adults. This research would provide information to help support or refute the sociocognitive theories surrounding emotional perception of speech. Another direction to be explored could examine the acoustic features of clear speech. By examining the Group A talkers, we could potentially identify speaking strategies that make speech clearer without perceived anger. When these acoustic strategies are found, informational materials could be created that educate audiologists and aural rehabilitation specialists to modify their counseling and training of frequent communication partners.

## Data Availability

The statistical analyses with accompanying figures for visualization are available via an online Rmarkdown file accessible at: https://rpubs.com/shaedmorgan/agingaffectsangerperception. Other data and materials used in this study are available upon request from the corresponding author.
